# Efficacy of capecitabine and oxaliplatin versus S-1 as adjuvant chemotherapy in gastric cancer after D2 lymph node dissection according to lymph node ratio and N stage

**DOI:** 10.1186/s12885-019-6433-3

**Published:** 2019-12-18

**Authors:** Kabsoo Shin, Se Jun Park, Jinsoo Lee, Cho Hyun Park, Kyo Young Song, Han Hong Lee, Ho Seok Seo, Yoon Ju Jung, Jae Myung Park, Sung Hak Lee, Sang Young Roh, In-Ho Kim

**Affiliations:** 1 Division of Medical Oncology, Department of Internal Medicine, Seoul St. Mary’s Hospital, College of Medicine, The Catholic University of Korea, 222 Banpo-daero, Seocho-gu, Seoul, 137-701 South Korea; 2 Department of Surgery, Seoul St. Mary’s Hospital, College of Medicine, The Catholic University of Korea, Seoul, South Korea; 3 Department of Gastric Cancer Centre, Seoul St. Mary’s Hospital, College of Medicine, The Catholic University of Korea, Seoul, South Korea; 4 Division of Gastroenterology, Department of Internal Medicine, Seoul St. Mary’s Hospital, College of Medicine, The Catholic University of Korea, Seoul, South Korea; 5 Department of Clinical Pathology, Seoul St. Mary’s Hospital, College of Medicine, The Catholic University of Korea, Seoul, South Korea; 6 Cancer Research Institute, College of Medicine, The Catholic University of Korea, Seoul, South Korea

**Keywords:** Tegafur, Capecitabine, Oxaliplatin, Gastric cancer, Lymph node ratios, N stage, Propensity score matching

## Abstract

**Background:**

We sought to assess the prognostic significance of lymph node ratio (LNR) and N stage in patients undergoing D2 gastrectomy and adjuvant chemotherapy, S-1, and XELOX and to compare the efficacy of them according to LNRs and N stages to evaluate the clinical impact of using LNRs compared with using N staging.

**Methods:**

Patients undergoing D2 gastrectomy with adequate lymph node dissection and adjuvant chemotherapy for stage II/III gastric cancer between Mar 2011 and Dec 2016 were analysed. Of the 477 patients enrolled, 331 received S-1 and 146 received XELOX. LNR groups were segregated as 0, 0–0.1, 0.1–0.25, and > 0.25 (LNR0, 1, 2, and 3, respectively). Propensity score matching (PSM) was used to minimise potential selection bias and compare DFS and OS stratified by LNRs and N stages in the two treatment groups.

**Results:**

After PSM, the sample size of each group was 110 patients, and variables were well balanced. All patients had more than 15 examined lymph nodes (median 51, range 16~124). In multivariate analysis, LNR (> 0.25) and N stage (N3) showed independent prognostic value in OS and DFS, but LNR (> 0.25) showed better prognostic value. In subgroup analysis, the LNR3 group showed better 5-year DFS (20% vs 54%; HR 0.29; *p* = 0.004) and 5-year OS (26% vs 67%; HR 0.28; *p* = 0.020) in the XELOX group. The N3 group showed better 5-year DFS (38% vs 66%; HR 0.40; *p* = 0.004) and 5-year OS (47% vs 71%; HR 0.45; *p* = 0.019) in the XELOX group. Stage IIIC showed better 5-year DFS (22% vs 57%; HR 0.32; p = 0.004) and 5-year OS (27% vs 68%; HR 0.32; *p* = 0.009) in the XELOX group. The LNR3 group within N3 patients showed better 5-year DFS (21% vs 55%; HR 0.31; *p* = 0.004) and 5-year OS (27% vs 68%; HR 0.34; *p* = 0.018) in the XELOX group.

**Conclusions:**

LNR showed better prognostic value than N staging. LNR3, N3 and stage IIIC groups showed the superior efficacy of XELOX to that of S-1. And the LNR3 group within N3 patients showed more survival benefit from XELOX. LNR > 0.25, N3 stage and stage IIIC were the discriminant factors for selecting XELOX over S-1.

**Trial registration:**

Not applicable (retrospective study).

## Background

Gastric cancer is the fifth most common cancer and the third leading cause of cancer death worldwide, accounting for over 1,000,000 newly diagnosed cancer patients and over 783,000 cancer-related deaths annually [1]. Radical gastrectomy with extended lymphadenectomy (D2 gastrectomy) is the standard of care for gastric cancer in many countries in East Asia [2, 3]. Although the safety and utility of extended lymph node dissection have been debated for a long time in Europe and the US, D2 gastrectomy is recommended based on several trials (especially the Dutch D1D2 study), which showed a reduction in cancer-related deaths with D2 gastrectomy [4–6].

However, the recurrence rate of D2 gastrectomy is high. Approximately 40% of patients relapse within 2 years of surgery, necessitating adjuvant treatment [7–9]. Adjuvant treatments for gastric cancer differ by geographical region. In the UK and other European countries, perioperative chemotherapy is recommended as a standard treatment [10]. In the USA, the recommended adjuvant therapy is postoperative chemoradiation or chemotherapy, depending on the type of lymph node dissection [11]. The evidence of postoperative chemoradiation is based on the UK Medical Research Council Adjuvant Gastric Infusional Chemotherapy (MAGIC) trial [12] and the US Intergroup-0116 trial [13]. Both studies assessed the survival benefits of adjuvant therapy after limited dissection of the regional lymph nodes.

The evidence of postoperative chemotherapy is based on two randomised controlled trials that investigated the efficacy of adjuvant chemotherapy after D2 gastrectomy compared to D2 gastrectomy alone in patients with resectable gastric cancer [2, 14]. In the ACTS-GC trial in Japan, patients with Stage II, III gastric cancer were treated with D2 gastrectomy, and showed a hazard ratio (HR) for 5-year overall survival (OS) of 0.669 [95% confidence intervals (CI), 0.540–0.828] in the comparison of 1) surgery and adjuvant chemotherapy treatment with oral fluoropyrimidine S-1 for 1 year versus 2) surgery alone and a 5-year follow-up. In the CLASSIC trial, which took place mainly in South Korea, patients with Stage II, III gastric cancer were treated with D2 gastrectomy, and showed an HR for 3-year disease-free survival (DFS) of 0.56 (95% CI, 0.44–0.72; *p* < 0.0001) and for OS of 0.72 (95% CI, 0.52–1.00; *p* = 0.049) in the comparison of 1) adjuvant capecitabine and oxaliplatin for 6 months after D2 gastrectomy versus 2) surgery alone after a median follow-up of 34 months [2, 14]. Despite this evidence, there has been no prospective study that directly compare S-1 and XELOX. Previous studies suggested that XELOX would be more beneficial for more aggressive disease with higher N stage [15, 16].

In addition to the TNM staging system, the ratio of positive and total examined lymph nodes (lymph node ratio, LNR) has been proposed as a simple and convenient tool for identifying subgroups of gastric cancer patients with similar prognosis. It can also be used to adjust stage migration from current tumour, node, metastasis (TNM) staging of gastric cancer. Cut-off values of 0.1 and 0.25 have been adopted in several studies and have been found to be in good agreement to the N1, N2, and N3 stages of the 6th and 7th UICC/TNM staging system [17–21]. However, the significance of LNR has not been evaluated for patients with adjuvant chemotherapy after D2 gastrectomy. Furthermore, whether LNR is more accurate prognostic and predictive than N stage is not clear in these patients.

Therefore, we sought to 1) assess the prognostic significance of LNR and N stage in patients undergoing D2 gastrectomy and adjuvant chemotherapy, S-1, and XELOX and 2) assess the efficacy of adjuvant S-1 and XELOX according to LNRs and N stages to evaluate the clinical impact of using LNRs compared with using N staging.

## Methods

### Patients

We retrospectively investigated the data of 798 patients who underwent curative resection for gastric cancer and diagnosed as stage II or III between Mar 2011 and Dec 2016 at the Catholic University of Seoul St. Mary’s hospital.

**Fig. 1:**
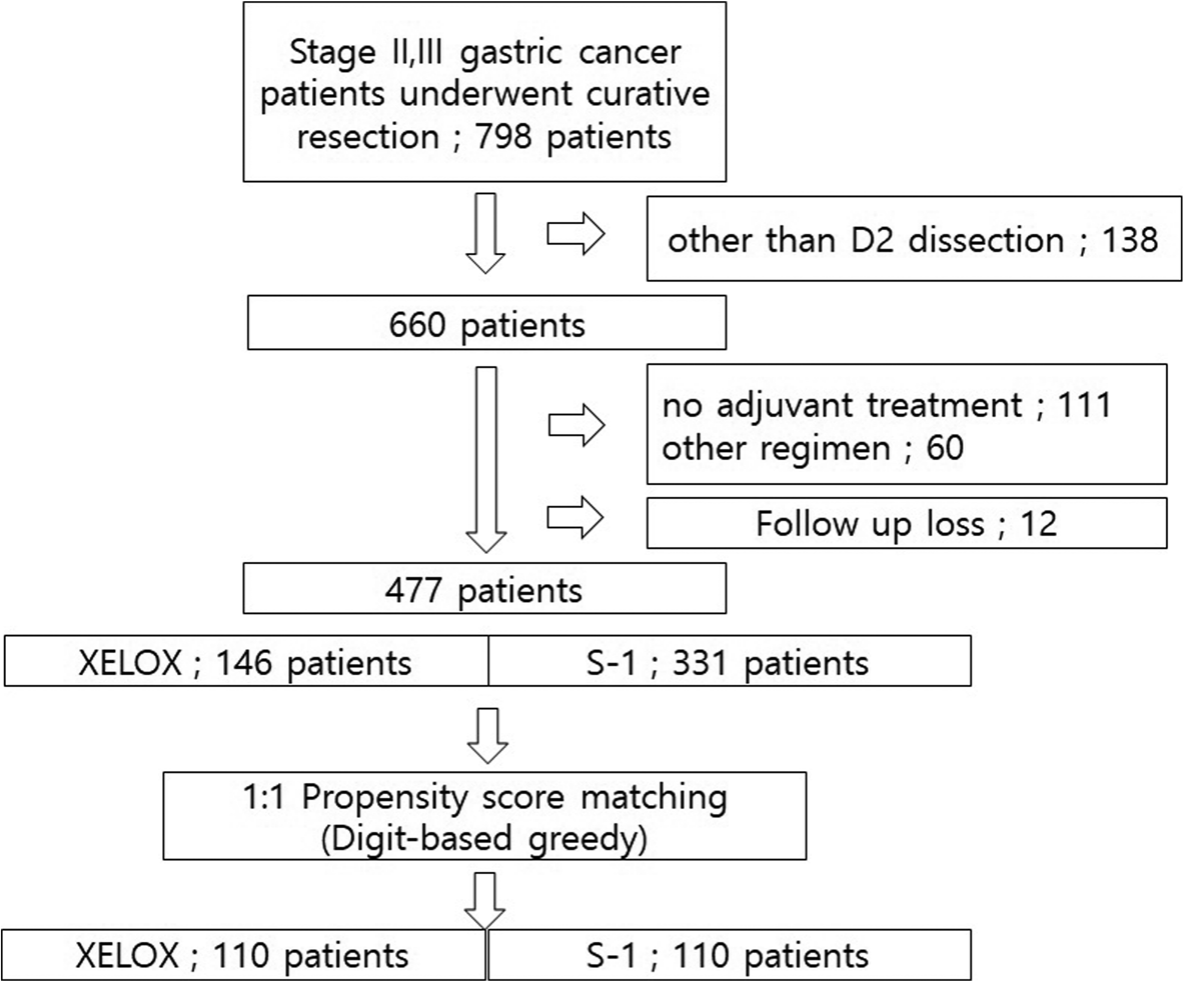
Study flow diagram according to the eligible criteria. After 321 of 798 patients were excluded, data from 477 patients were analysed retrospectively. The propensity score matching was performed between XELOX group and S-1 group

Among these patients, eligible patients (1) were aged 18 years or older, (2) had histologically confirmed gastric adenocarcinoma after radical gastrectomy with D2 lymph node dissection and R0 resection (3) had stage II or III disease (based on the 7th edition of the American Joint Committee on Cancer criteria) and (4) had no prior treatment for cancer other than the initial gastric resection for the primary lesion. After 321 of 798 patients were excluded, 477 met the eligibility criteria and received XELOX or S-1. (Fig. 1).

Patients in the XELOX group received oral capecitabine (1000 mg/m^2^ twice daily (on days 1–14 of each cycle) plus intravenous oxaliplatin (130 mg/m^2^ on day 1 of each cycle) every 3 weeks. The duration of XELOX was eight cycles (6 months). Patients in the S-1 group received daily doses of 80 mg, 100 mg or 120 mg of S-1. Those with a body-surface area of less than 1.25 m^2^ received 80 mg daily; those with a body-surface area of 1.25 m^2^ or more but less than 1.5 m^2^ received 100 mg daily; and those with a body-surface area of 1.5 m^2^ or more received 120 mg daily. In each six-week cycle, S-1 was administered for 4 weeks, followed by a two-week resting period. The duration of S-1 was eight cycles (12 months).

The Institutional Review Board of the Catholic University of Seoul Saint Mary’s Hospital approved the study (KC18RESI0596, KC19RASI0751). Requirement for informed consent was waived because the study was based on retrospective analyses of existing administrative and clinical data.

### Follow-up evaluation

Tumour assessments were performed with abdominal computed tomography (CT) or magnetic resonance imaging (MRI) every two or three cycles of treatment with tumour marker; CEA, CA 19–9. After finishing adjuvant chemotherapy, tumour assessments were performed every 6 months for the first 3 years and yearly thereafter. When signs or symptoms indicated a possible recurrence or development of new gastric cancer, additional imaging or biopsies were performed to confirm the presence of malignancy.

Disease-free survival (DFS) was defined as the interval between the time from the curative resection of gastric cancer until the date of disease recurrence at locoregional and/or distant sites, or the date of death from any cause. Overall survival (OS) was measured as the time from the curative resection of the gastric cancer until death from any cause or until the last follow-up date.

### Statistical analyses

To directly compare the efficacies of S-1 and XELOX chemotherapies, DFS and OS were determined and 5-year DFS and 5-year OS were compared. To minimise the influence of potential confounders on selection bias, propensity score matching (PSM) was performed. The propensity scores were elicited from matched patients at 1:1 ratio using greedy matching algorithms without replacement. Age, sex, ECOG (**Eastern Cooperative Oncology Group)** performance status, ASA (American Society of Anesthesiologists) score, location of the tumour, stage (based on the 7th AJCC guidelines), T stage, N stage, number of dissected lymph nodes, tumour size, LNR group, differentiation, Lauren classification, lymphovascular invasion, perineural invasion, completion of planned chemotherapy, preoperative CEA and CA 19–9 were used to calculate propensity scores for each patient using logistic regression. Standardized differences were estimated for all covariates before and after matching to assess pre-match imbalance and post-match balance.

A Wilcoxon rank sum test for continuous variables or Chi-square test for categorical variables was used to compare the demographics between treatment arms in before PSM data. A Wilcoxon signed rank sum test for continuous variables or Chi-square test for categorical variables was used in matched data. The Kaplan-Meier method was used to estimate cumulative survival. The treatment groups were compared with a two-sided log-rank test. Estimates of treatment effect were calculated with 95% Cis using Cox proportional hazards models.

Univariate and multivariate analysis models of patient and tumour characteristics in association with DFS and OS were based on Cox-proportional hazards regression analyses. *P* values of less than 0.05 were considered to indicate statistical significance. All statistical analyses were conducted using SAS software ver. 9.4 (SAS Institute Inc., Cary, NC, USA) and R version 3.5.3 (http://www.r-project.org).

## Results

### Clinical characteristics

Of the 477 patients eligible for this study, 331 received S-1 and 146 received XELOX. The median age was 57 years (range 22 ~ 79), and the male: female ratio was 326 (68.3%): 151 (31.7%). The median follow-up duration was 52.3 months. The baseline characteristics of the patients in the two groups are summarised in Table 1. Before PSM, the two groups differed significantly in age, ECOG performance status, cancer stage (AJCC 7th edition), T stage, N stage, number of dissected lymph nodes, LNR group, tumour size, differentiation, lymphovascular invasion, perineural invasion.

**Table 1:** Baseline characteristics of the patients before and after propensity score matching

	Before propensity score matching (*n* = 477)				After propensity score matching §(*n* = 220)			
	S-1 (*n* = 331)	XELOX (*n* = 146)	*p* value*	Absolute‡ Standardized difference in %	S-1 (*n* = 110)	XELOX (*n* = 110)	*p* value†	Absolute‡ Standardized difference in %
Age (years)								
< 65	181 (54.7)	104 (71.2)	0.001	34.7	70 (63.6)	68 (61.8)	0.889	3.7
≥ 65	150 (45.3)	42 (28.8)		34.7	40 (36.4)	42 (38.2)		3.7
Sex								
Male	225 (68.0)	101 (69.2)	0.795	2.6	76 (69.1)	76 (69.1)	> 0.999	< 0.001
Female	106 (32.0)	45 (30.8)		2.6	34 (30.9)	34 (30.9)		< 0.001
ECOG								
0	241 (72.8)	124 (84.9)	0.004	30.0	86 (78.2)	89 (80.9)	0.738	6.7
≥ 1	90 (27.2)	22 (15.1)		30.0	24 (21.8)	21 (19.1)		6.7
ASA score								
1 to 2	308 (93.1)	138 (94.5)	0.549	3.4	100 (90.9)	103 (93.6)	0.615	9.2
≥ 3	23 (6.9)	8 (5.5)		3.4	10 (9.1)	7 (6.4)		9.2
Location								
EGJ	11 (3.3)	7 (4.8)	0.437	7.4	107 (97.3)	108 (98.2)	> 0.999	6.1
Other	320 (96.7)	139 (95.2)		7.4	3 (2.7)	2 (1.8)		6.1
Stage (AJCC 7th edition)								
IIA	109 (32.9)	5 (3.4)	< 0.001	82.8	3 (2.7)	4 (3.6)	0.982	5.2
IIB	73 (22.1)	19 (13.0)		23.9	21 (19.1)	19 (17.3)		4.7
IIIA	52 (15.7)	39 (26.7)		27.2	28 (25.5)	26 (23.6)		4.2
IIIB	53 (16.0)	48 (32.9)		40.0	33 (30.0)	34 (30.9)		2.0
IIIC	44 (13.3)	35 (24.0)		27.7	25 (22.7)	27 (24.5)		4.3
T stage								
T1	26 (7.9)	3 (2.1)	0.001	27.0	4 (3.6)	3 (2.7)	> 0.999	5.2
T2	51 (15.4)	10 (6.8)		27.5	8 (7.3)	8 (7.3)		0.0
T3	129 (39.0)	56 (38.4)		1.3	40 (36.4)	40 (36.4)		0.0
T4a,b	125 (37.8)	77 (52.7)		30.4	58 (52.7)	59 (53.6)		1.8
N stage								
N0	87 (26.3)	9 (6.2)	< 0.001	56.7	9 (8.2)	9 (8.2)	0.986	0.0
N1	67 (20.2)	28 (19.2)		2.7	16 (14.5)	17 (15.5)		2.5
N2	103 (31.1)	38 (26.0)		11.3	39 (35.5)	36 (32.7)		5.8
N3	74 (22.4)	71 (48.6)		57.1	46 (41.8)	48 (43.6)		3.7
Number of dissected lymph nodes								
mean ± sd	47.0 ± 18.8	52.4 ± 17.1	< 0.001	30.0	51.4 ± 21.4	51.5 ± 16.5	0.493	0.7
median (IQR)	43 (35–55)	52 (39–65)			45 (37–64)	52 (39–62)		
LNR group								
LNR 0	88 (26.6)	9 (6.2)	< 0.001	40.2	68 (61.8)	66 (60.0)	0.89	3.7
LNR 1	127 (38.4)	49 (33.6)		40.2	42 (38.2)	44 (40.0)		3.7
LNR 2	78 (23.6)	47 (32.2)						
LNR 3	38 (11.5)	41 (28.1)	< 0.001	57.4	9 (8.2)	9 (8.2)	0.994	0.0
Tumor size (cm)				10.0	36 (32.7)	35 (31.8)		1.9
< 6	250 (75.5)	83 (56.8)		19.3	39 (35.5)	41 (37.3)		3.8
≥ 6	81 (24.5)	63 (43.2)		42.6	26 (23.6)	25 (22.7)		2.2
Differentiation								
Well to moderately	114 (34.4)	34 (23.3)	0.015	24.8	25 (22.7)	28 (25.5)	0.753	6.4
Poorly	217 (65.6)	112 (76.7)		24.8	85 (77.3)	82 (74.5)		6.4
Lauren classification								
Intestinal	118 (35.6)	39 (26.7)	0.111	19.4	30 (27.3)	34 (30.9)	0.732	8.0
Diffuse	96 (29.0)	43 (29.5)		1.0	37 (33.6)	32 (29.1)		9.8
Mixed	117 (35.3)	64 (43.8)		17.4	43 (39.1)	44 (40.0)		1.9
Lymphovascular invasion								
no	90 (27.2)	13 (8.9)	< 0.001	50.2	8 (7.3)	13 (11.8)	0.359	8.9
yes	241 (72.8)	133 (91.1)		50.2	102 (92.7)	97 (88.2)		8.9
Perineural invasion								
no	161 (48.6)	49 (33.6)	0.002	30.9	40 (36.4)	39 (35.5)	> 0.999	1.9
yes	170 (51.4)	97 (66.4)		30.9	70 (63.6)	71 (64.5)		1.9
Completion of planned chemotherapy								
no	69 (20.8)	42 (28.8)	0.059	18.4	25 (22.7)	26 (23.6)	> 0.999	2.1
yes	262 (79.2)	104 (71.2)		18.4	85 (77.3)	84 (76.4)		2.1
CEA (ng/ml)								
< 5	315 (95.2)	140 (95.9)	0.728	3.5	106 (96.4)	106 (96.4)	> 0.999	< 0.001
≥ 5	16 (4.8)	6 (4.1)		3.5	4 (3.6)	4 (3.6)		< 0.001
CA 19–9 (U/ml)								
< 37.0	308 (93.1)	132 (90.4)	0.320	9.6	100 (90.9)	102 (92.7)	0.806	9.7
≥ 37.0	23 (6.9)	14 (9.6)		9.6	10 (9.1)	8 (7.3)		9.7

Data are presented as the n (%) for categorical variable, unless otherwise indicated

*P value from Wilcoxon rank sum test for continuous variables or Chi-square test, for categorical variables in before Propensity score matching data

†*P* value from Wilcoxon signed rank sum test for continuous variables or Chi-square test, for categorical variables in matched data

‡no covariates would be considered imbalanced if the threshold was set at either 0.10 (Normand et al. 2001) or 0.25 (Rubin 2001)

§matched using digit-based greedy (“greedy”)

The XELOX group had a younger age than the S-1 group (S-1 vs XELOX, median age 58 vs 55 years, *p* < 0.001). The XELOX group had a smaller number of patients aged more than 65 years than the S-1 group (S-1 vs XELOX, 45.3% vs 28.8%, *p* = 0.001). The XELOX group had a smaller number of patients with ECOG PS ≥ 1 than the S-1 group (S-1 vs XELOX, 27.2% vs 15.1%, *p* = 0.004). Compared with the S-1 group, the XELOX group had patients with more advanced T and N stages of gastric cancer (p = 0.001, < 0.001 respectively), had patients with an increased number of dissected lymph nodes (S-1 vs XELOX, median (IQR) 43(35–55) vs 52(39–65), *p* < 0.001), and had a greater number of patients in the higher LNR groups (median LNR 0.06 vs 0.13, *p* < 0.001).

An increased number of patients with tumour size (≥6 cm) was observed in the XELOX group compared to the S-1 group (S-1 vs XELOX, 24.5% vs 43.2%, *p* < 0.001). The percentage of patients assigned a ‘poorly differentiated’ histologic grade was also higher in the XELOX group than in the S-1 group (S-1 vs XELOX, 65.6% vs 76.7% *p* = 0.015).

Lymphovascular invasion and perineural invasion were more significantly more frequently observed in the XELOX group than in the S-1 group (S-1 vs XELOX, 72.8% vs 91.1, 51.4% vs 66.4%, respectively). The rate of chemotherapy completion in the S-1 group showed tendency to be higher than that in the XELOX group (S-1 vs XELOX, 79.2% vs 71.2%, *p* = 0.059). After PSM, each group was one-to-one matched so that there were 110 patients per group. Each variable was well balanced, without significant difference in terms of absolute standardised difference (Table 1).

### Univariate and multivariate analyses of DFS and OS in the PSM cohort. (Table 2)

Upon univariate analysis of all patients after PSM, age (< 65 vs ≥65), ECOG performance status (0 vs ≥1), N stage (N0,1,2 vs N3), LNR group (LNR0,1,2 vs LNR3), tumour size (≥6 cm), lymphovascular invasion, perineural invasion, and completion of planned chemotherapy were shown as prognostic factors associated with survival. After adjusting for covariates in multivariate analysis, N stage (HR 1.40; 95% CI, 1.09–1.80; *p* = 0.009), LNR group (HR 1.36; 95% CI, 1.09–1.70; *p* = 0.006), perineural invasion (HR 2.39; 95% CI, 1.18–4.82; *p* = 0.015) and completion of planned chemotherapy(HR 0.50; 95% CI, 0.28–0.91; *p* = 0.023) were shown as independent prognostic factors of survival.

**Table 2:** Univariate, multivariate cox proportional hazards regression in the PSM cohort. (*n* = 220)

	Overall survival						Disease-free survival					
	univariate			multivariate			univariate			multivariate		
	HR	(95%CI)	*p* value	HR	(95%CI)	p value	HR	(95%CI)	*p* value	HR	(95%CI)	*p* value
Treatment												
S-1	1						1					
XELOX	0.71	0.40–1.26	0.240				0.65	0.39–1.09	0.101			
Age (years)												
< 65	1			1			1					
≥ 65	1.93	1.11–3.35	**0.02**	1.33	0.72–2.46	0.363	1.58	0.96–2.60	0.07			
Sex												
Female	1						1					
Male	1.14	0.64–2.04	0.66				1.25	0.85–2.11	0.393			
ECOG												
0	1			1			1			1		
≥ 1	2.32	1.31–4.12	**0**	1.54	0.80–3.00	0.198	2.17	1.28–3.66	**0**	1.72	0.99–2.98	0.051
ASA												
1 to 2	1						1					
≥ 3	0.85	0.58–1.26	0.420				1.1	0.85–1.43	0.462			
Location												
Other	1						1					
EGJ	0.54	0.13–2.23	0.398				2.82	0.88–9.01	0.081			
T stage												
T1,T2	1						1					
T3,T4	3.19	0.78–13.12	0.108				2.65	0.83–8.47	0.1			
N stage												
N0,1,2	1	1		1			1			1		
N3	1.69	1.37–2.09	**< 0.001**	1.4	1.09–1.80	**0.009**	1.54	1.29–1.84	**< 0.001**	1.26	1.00–1.58	**0.05**
LNR group												
LNR0,1,2	1			1			1			1		
LNR3	1.7	1.41–2.04	**< 0.001**	1.36	1.09–1.70	**0.006**	1.67	1.41–1.97	**< 0.001**	1.44	1.16–1.78	**0**
Tumor size												
< 6	1			1			1			1		
≥ 6	1.95	1.13–3.39	**0.02**	1.07	0.97–1.18	0.209	1.91	1.16–3.13	**0.01**	1.049	0.96–1.15	0.288
Differntiation												
Well to moderately	1						1					
Poorly	0.81	0.44–1.51	0.512				0.95	0.53–1.69	0.855			
Lauren classification												
Intestinal	1						1					
Diffuse/Mixed	0.94	0.70–1.26	0.681				0.99	0.75–1.30	0.923			
Lymphovascular invasion												
no	1						1					
yes	3.13	0.76–12.88	0.114				2.53	0.79–8.07	0.117			
Perineural invasion												
no	1			1			1			1		
yes	2.72	1.36–5.43	**0.01**	2.39	1.18–4.82	**0.015**	2.05	1.15–3.66	**0.02**	1.47	0.81–2.66	0.205
Chemotherapy completion												
no	1			1			1			1		
yes	0.43	0.24–0.77	**0**	0.5	0.28–0.91	**0.023**	0.36	0.21–0.59	**< 0.001**	0.36	0.21–0.61	**< 0.001**
CEA (before surgery)												
normal	1						1					
elevated	1.31	0.32–5.38	0.711				1.02	0.25–4.19	0.975			
CEA (after surgery)												
normal	1						1					
elevated	1.14	0.28–4.68	0.86				0.91	0.22–3.74	0.902			
CA 19–9 (before surgery)												
normal	1						1			1		
elevated	1.87	0.84–4.16	0.123				2.66	1.36–5.24	**0.01**	1.81	0.88–3.74	0.107
CA 19–9 (after surgery)												
normal	1						1					
elevated	1.32	0.32–5.41	0.705				2.09	0.65–6.67	0.213			

Univariate analysis and multivariate survival analysis were performed using Cox proportional hazard model, and P values < 0.05 were considered to indicate statistical significance

Abbreviations: *CI* confidence interval, *HR* hazard ratio. Significant values are in boldface type

In addition, ECOG performance status (0 vs ≥1), N stage (N0,1,2 vs N3), LNR group (LNR0,1,2 vs LNR3), tumour size (≥6 cm), perineural invasion, completion of planned chemotherapy, and elevated preoperative CA 19–9 were shown as prognostic factors associated with recurrence. After adjusting for covariates in multivariate analysis, N3 stage (HR 1.26; 95% CI, 1.00–1.58; *p =* 0.049), LNR3 group (HR 1.44; 95% CI, 1.16–1.78; *p* = 0.001), and completion of planned chemotherapy (HR 0.36; 95% CI, 0.21–0.61; *p <* 0.001) were shown as independent prognostic factors of recurrence.

### Subgroup analysis of the PSM cohort. S-1 vs XELOX

**Fig. 2:**
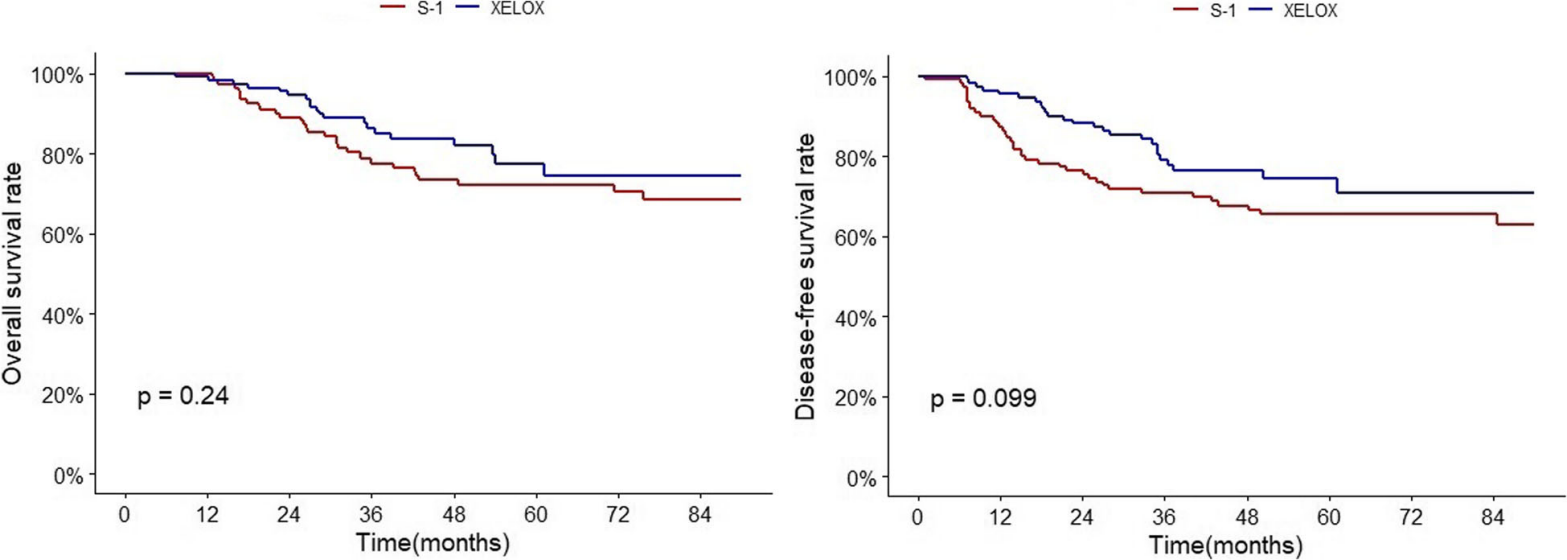
OS and DFS of S-1 and XELOX in the PSM cohort

After PSM, OS and DFS were higher in the XELOX group than in the S-1 group, with HR of 0.71 (95% CI 0.40–1.26; *p* = 0.240) and 0.65 (95% CI 0.39–1.09; *p* = 0.101). The 5-year DFS rate in the S-1 group versus the XELOX group was 66% versus 74%. The 5-year OS rate in the S-1 vs XELOX groups was 72% versus 77%. Both DFS and OS rates were not significantly different between the two groups. (Table 3, Fig. 2).

**Table 3:** DFS, OS of XELOX and S-1 in the PSM cohort

	total	event	3 year	5 year	HR(95% CI)^a^	*p* value
Ovarall survival			3-year OS % (95% CI)	5-year OS % (95% CI)		
TS-1	110	31	78 (70–86)	72 (64–81)	1	0.240
XELOX	110	20	86 (80–93)	77 (68–88)	0.71 (0.40–1.26)	
Disease-free survival			3-year DFS % (95% CI)	5-year DFS % (95% CI)		
TS-1	110	38	71 (63–80)	66 (57–75)	1	0.101
XELOX	110	25	79 (72–88)	74 (66–84)	0.65 (0.39–1.09)	

^a^HR of XELOX adjuvant chemotherapy for recurrence of gastric cancer compared with S-1 as the reference was calculated using Cox’s proportional hazards model

Abbreviations: *CI* confidence interval, *HR* hazard ratio. Significant values are in boldface type

**Fig. 3:**
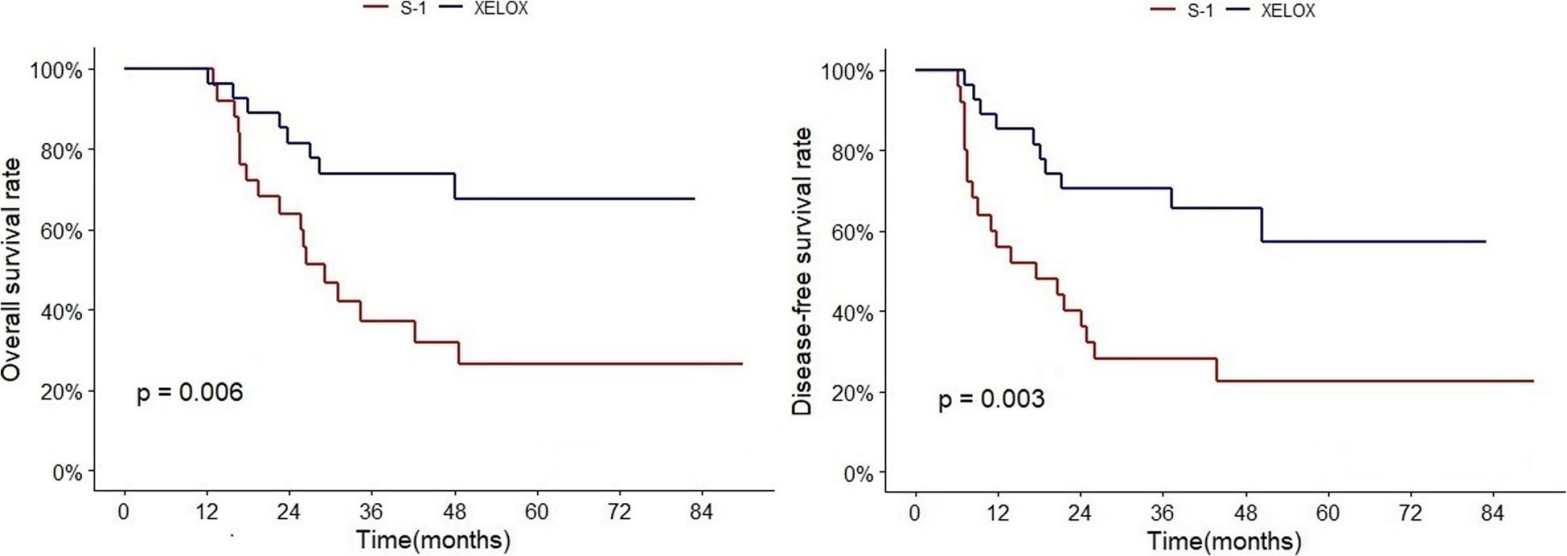
OS and DFS of XELOX and S-1 in Stage IIIC. XELOX regimen showed significantly better efficacy compared to S-1 in Stage IIIC patients in terms of OS and DFS

Subgroup analysis of the PSM data set revealed that the XELOX group, compared with the S-1 group, showed significantly better 5-year DFS (S-1 vs XELOX, 22% vs 57%, HR 0.32, 95% CI 0.15–0.70; *p* = 0.004) and better 5-year OS (27% vs 68%, HR 0.32, 95% CI 0.14–0.76; *p* = 0.009) in stage IIIC patients. All stage III patients showed better DFS and OS in the XELOX group than in the S-1 group, but statistically not significant. (DFS 60% vs 69%, OS 67% vs 73%). (Table 4, Fig. 3, Additional file 1; survival curves of XELOX and S-1 in Stage IIIA, B, C).

**Table 4:** Subgroup analysis of the PSM cohort (*n* = 220)

	number of patients	Overall survival				Disease-free survival			
		5-year OS % (95% CI)		HR(95% CI)	*p* value*	5-year DFS % (95% CI)		HR(95% CI)	*p* value*
		S-1	XELOX			S-1	XELOX		
Sex									
Male	152	73 (63–84)	78 (68–90)	0.63 (0.32–1.27)	0.196	66 (56–78)	77 (67–88)	0.60 (0.32–1.14)	0.117
Female	68	71 (57–89)	76 (59–97)	0.93 (0.35–2.48)	0.890	64 (49–83)	68 (49–94)	0.75 (0.32–1.77)	0.507
Age (years)									
< 65	138	78 (68–88)	87 (79–96)	0.66 (0.29–1.50)	0.316	71 (61–83)	77 (67–90)	0.73 (0.36–1.44)	0.361
≥ 65	82	62 (48–80)	64 (48–85)	0.69 (0.32–1.51)	0.358	56 (42–74)	69 (55–87)	0.55 (0.261–1.18)	0.125
Stage (AJCC 7th)									
IIA	7	100 (100–100)	100 (100–100)	NA	NA	100 (100–100)	100 (100–100)	NA	NA
IIB	40	89 (64–97)	92 (57–99)	0.67 (0.06–7.48)	0.747	85 (61–95)	92 (54–99)	0.38(0.04–3.68)	0.405
IIIA	54	89 (70–96)	75 (33–93)	1.56 (0.31–7.96)	0.593	85 (66–94)	77 (53–90)	1.58(0.43–5.76)	0.487
IIIB	67	78 (65–94)	72 (55–94)	1.35 (0.50–3.69)	0.554	66 (51–85)	74 (60–92)	0.84 (0.34–2.04)	0.697
IIIC	52	27 (10–46)	68 (51–90)	0.32 (0.14–0.76)	**0.009**	22 (8–41)	57 (39–84)	0.32 (0.15–0.70)	**0.004**
All II	47	90 (78–100)	94 (83–100)	0.58 (0.05–6.40)	0.655	87(74–100)	93 (82–100)	0.35 (0.04–3.34)	0.360
All III	173	67 (58–78)	73 (62–86)	0.73 (0.40–1.31)	0.285	60 (50–71)	69 (59–81)	0.67 (0.40–1.13)	0.133
N stage									
N0	18	100 (100–100)	100 (100–100)	NA	NA	100 (100–100)	100 (100–100)	NA	NA
N1	33	93 (59–99)	81 (52–94)	3.40 (0.35–32.86)	0.290	87 (56–96)	80 (50–93)	1.81(0.30–10.96)	0.519
N2	75	86 (71–94)	77 (42–92)	1.40 (0.36–5.41)	0.623	82 (65–91)	78 (57–90)	1.18(0.42–3.34)	0.757
N3	94	47 (34–65)	71 (58–86)	0.45 (0.23–0.87)	**0.019**	38 (26–55)	66 (52–82)	0.40 (0.21–0.75)	**0.004**
T stage									
T1	7	100 (100–100)	100 (100–100)	NA	NA	75 (13–96)	100 (100–100)	0.42(0.00–41.43)	0.712
T2	16	86 (33–98)	86 (33–98)	0.87 (0.05–13.85)	0.919	88 (39–98)	86 (33–98)	0.93(0.06–14.83)	0.957
T3	80	80 (68–93)	81 (66–99)	0.75 (0.25–2.23)	0.604	74 (62–90)	80 (67–95)	0.78 (0.30–2.05)	0.617
T4a,b	117	64 (52–78)	72 (59–88)	0.69 (0.35–1.37)	0.290	56 (42–68)	67 (50–79)	0.61 (0.33–1.14)	0.121
LNR group									
LNR 0	18	100 (100–100)	100 (100–100)	NA	NA	100 (100–100)	100 (100–100)	NA	NA
LNR 1	71	94 (78–98)	86 (61–96)	3.01 (0.55–16.59)	0.205	88 (72–95)	85 (65–94)	1.43(0.41–4.99)	0.579
LNR 2	80	74 (57–85)	72 (50–86)	0.84 (0.34–2.10)	0.705	66 (49–79)	74 (56–85)	0.73(0.32–1.68)	0.464
LNR 3	51	26 (12–55)	67 (50–89)	0.28 (0.11–0.71)	**0.020**	20 (9–47)	54 (35–82)	0.29 (0.13–0.65)	**0.004**

*The hazard ratio of the XELOX group using the S-1 group as the reference and the 95% CIs were calculated using Cox’s proportional hazards model

†NA = not evaluable

Abbreviations: *CI* confidence interval, *HR* hazard ratio. Significant values are in boldface type

**Fig. 4:**
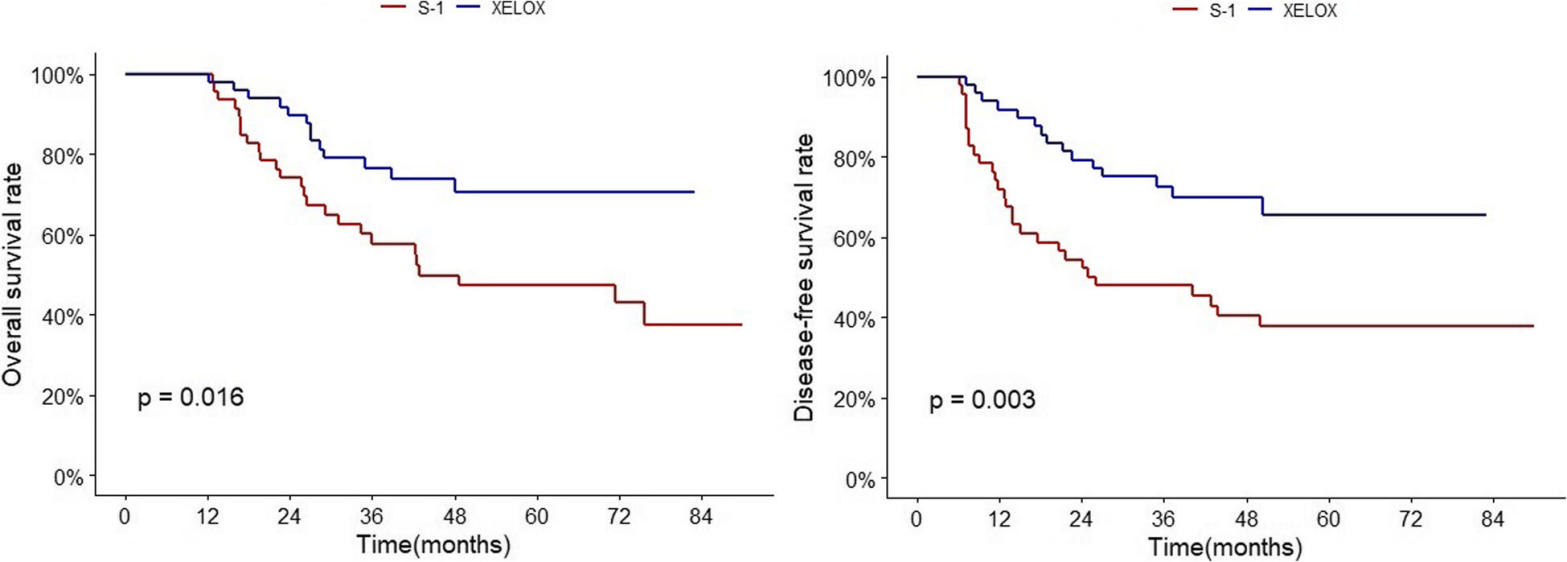
OS and DFS of XELOX and S-1 in N3. XELOX regimen showed significantly better efficacy compared to S-1 in N3 patients in terms of OS and DFS

When stratified by N stage in the PSM cohort, the XELOX group showed no difference in OS and DFS compared to the S-1 group in the N0, N1, and N2 groups. The N3 group showed significantly better 5-year DFS (38% vs 66%, HR 0.40, 95% CI 0.21–0.75; *p* = 0.004) and better 5-year OS (47% vs 71%, HR 0.45, 95% CI 0.23–0.87; *p* = 0.019) in the XELOX group (Table 4, Fig. 4, Additional file 2; survival curves of XELOX and S-1 in N1, 2, 3).

**Fig. 5:**
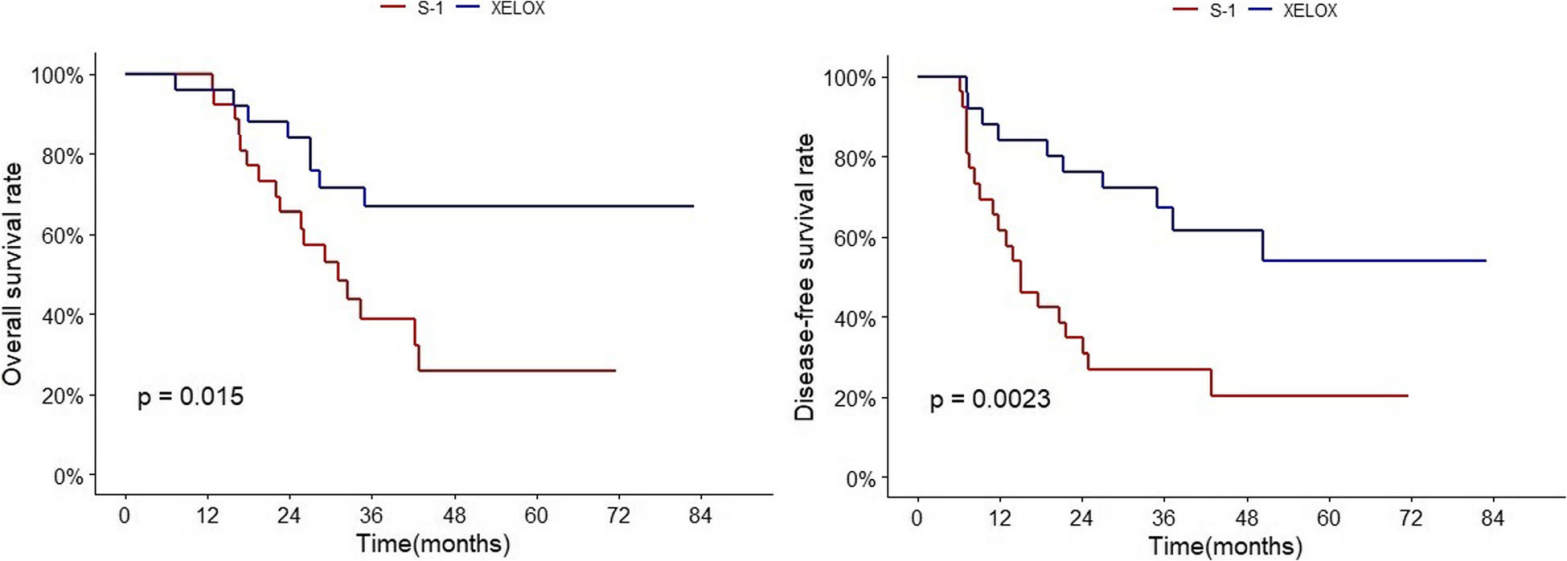
OS and DFS of XELOX and S-1 in LNR3. XELOX regimen showed significantly better efficacy compared to S-1 in LNR3 patients in terms of OS and DFS

When stratified by LNR group, LNR0, 1, 2 showed no significant difference in OS and DFS between the two regimens. The LNR3 group showed significantly better 5-year DFS in the XELOX group (20% vs 54%, HR 0.29, 95% CI 0.13–0.65; *p* = 0.004). The 5-year OS was also statistically different (26% vs 67%, HR 0.28, 95% CI 0.11–0.71; *p* = .0.020) (Table 4, Fig. 5, Additional file 3; survival curves of XELOX and S-1 in LNR1, 2, 3).

## Discussion

In this study, we analysed clinical impact of LNRs and N stages as prognostic factors and as clinical determinants for selecting XELOX or S-1 in the PSM cohort of gastric cancer patients after D2 gastrectomy with adequate lymph node dissection.

Perineural invasion was independent prognostic factors for survival consistent with previous studies that showed prognostic factors of gastric cancer [22]. N3, LNR3 and completion of planned chemotherapy showed the prognostic significance for both survival and recurrence.

Nitti et al. proposed a four-tier categorisation for N ratio (0, 1%~ 9, 10%~ 25, and > 25%) in gastric cancer, and reported that N ratio was an independent predictor of survival in their series [19]. Marchet et al. deduced the same conclusion with their Italian study [20]. Further, categorisation by N ratio has previously been utilised in clinical trials. Especially, the ARTIST trial compared XPRT with XP, and showed that XPRT was better in patients who had an N ratio of > 25% [23].

In this study, cut-off values of 0.1 and 0.25 have been adopted for categorizing four tiers of LNRs from Nitti’s study. The cut-off value for discriminating LNR3 from others was 0.25, which is similar to the 0.26 value calculated by a maximal chi-square method to identify optimal cutting point to discriminate all the PSM cohort patients into poor- and good-prognosis subgroups in terms of DFS [24]. And all the PSM cohort in this study underwent D2 gastrectomy, with more than 15 lymph nodes were examined (median 51, range 16~124), which is relatively higher than that examined in previous studies that showed prognostic value of LNR [21]. Although LNR is considered to have more prognostic value when the number of examined lymph nodes is less than 15, several studies showed that LNR has prognostic value regardless of retrieved lymph node and the LNR3 group in this study showed more prognostic value compared to N3 stage in both recurrence and survival in multivariate analysis [25–27].

In the N3 group, XELOX showed significant benefit for DFS and OS. This is consistent with the result of the CLASSIC trial and ACT-GC trial. The former showed a greater benefit in patients with node positive disease than in those whose disease was limited to N0, and the latter showed a minimal or no benefit when positive lymph node was equal to or more than three, even though they were deduced from subgroup analysis [2, 14].

**Fig. 6:**
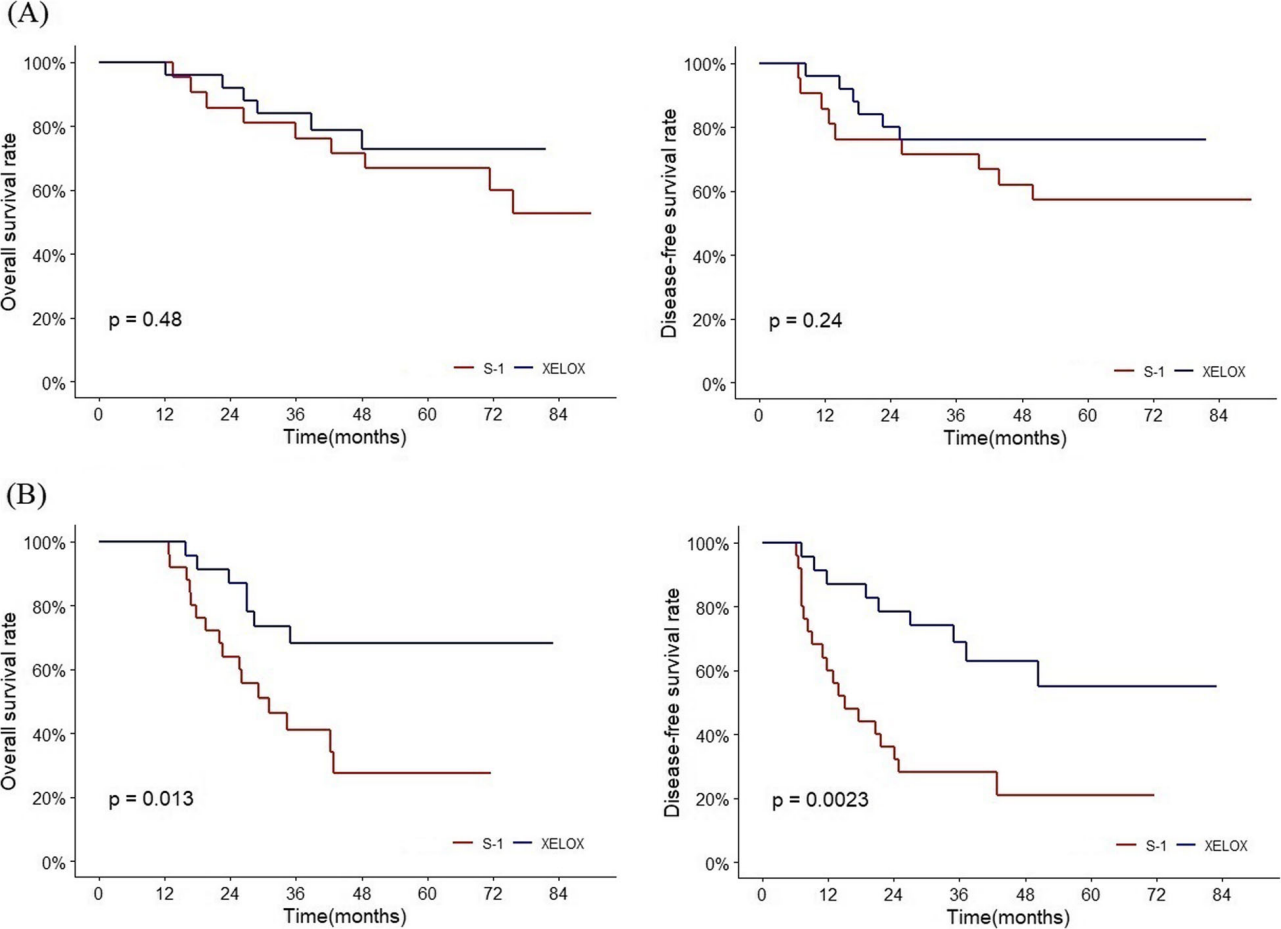
OS and DFS of XELOX and S-1 within N3. (A) LNR1,2 within N3. (B) LNR3 within N3. XELOX regimen showed significantly better efficacy compared to S-1 in LNR3 within N3, but not in LNR1,2 within N3

In the PSM cohort, the number of LNR3 patients were 51 (23.2%) and 48 of them classified to the N3 stage. (Table 5) When N3 group was divided into two groups; LNR3 group and LNR1,2 group, the XELOX and the S-1 in LNR1,2 group didn’t show difference in OS and DFS. However, LNR3 within N3 stage still showed significant survival benefit of the XELOX regimen (5-year DFS 21% vs 55% and 5-year OS 27% vs 68%, Fig. 6) This indicated that LNR3 can distinguish patients who can be more beneficial with XELOX regimen from N3 patients. Thus, for selecting XELOX or S-1, LNRs might have more clinical impact than N3 stage. However, its usefulness in patients with limited lymph node evaluation (examined LN ≤ 15) needs to be investigated further.

**Table 5:** The distribution of the lymph node ratio and N stage in the PSM cohort

	LNR0	LNR1	LNR2	LNR3	total
N stage					
N0	18				18
N1		31	2		33
N2		39	33	3	75
N3		1	45	48	94
total	18	71	80	51	220

Additionally, when stratified by stage (AJCC 7th edition) in the subgroup analysis of the PSM cohort, the XELOX group showed better DFS in stage IIIC patients. This result is consistent with that of a previous multi-centred, retrospective PSM study that compared XELOX and S-1. In the study, Kim et. all showed that XELOX was statistically more beneficial than S-1 in terms of 3-year DFS in stage IIIB, IIIC, and all stage III sub-types [15]. However, our study did not show the difference in DFS between the two regimens in stage IIIB and all stage III. The reason is that the sample size was too small to show statistical power. In the study, the 3-year DFS for S-1 vs XELOX in stage IIIB was 65.8% (95% CI, 61.2–70.4) vs 68.6% (95% CI, 55.9–81.3) (*p* = 0.019), and stage IIIB patients were 126 for S-1 and 48 for XELOX. Such a slim yet statistically significant difference might be explained by the relatively small sample size of this study, which included 33 patients for S-1 and 34 patients for XELOX in stage IIIB. And all stage III patients were 469 for Kim et al.‘s study and 173 patients for this study. Furthermore, our study showed that the XELOX group showed significantly better OS in stage IIIC, compared to the S-1 group.

This study had several limitations. Because this study used retrospective, single-centre data, it had the limitation of selection bias. Despite several efforts to reduce selection bias, including using multivariable analyses and PSM, unadjusted bias may have still been present between the two groups. Even though this study included as many clinical variables as possible in propensity matching, unmeasured variables might have still existed, resulting in unadjusted bias.

Moreover, this study only included patients with adjuvant chemotherapy. Thus, prognosis of the patients in this study should be interpreted with caution. Furthermore, a relatively small number of stage IIA (7 patients, 3.2% of the PSM cohort) was included in the PSM cohort even though their baseline characteristics were well-balanced after PSM.

## Conclusion

In gastric cancer patients underwent D2 gastrectomy with adequate lymph node dissection and adjuvant chemotherapy, LNR showed better prognostic value than N staging. Stage IIIC, LNR3 and N3 groups showed the superior efficacy of XELOX to that of S-1 in terms of DFS and OS. And the LNR3 group within N3 patients showed more survival benefit from XELOX. It suggests that using LNR might be useful for selecting patients for adjuvant chemotherapy regimens. LNR > 0.25, N3 stage and stage IIIC were the discriminant factors for selecting XELOX over S-1.

## Supplementary information

**Additional file 1: Figure S1.** DFS and OS of XELOX and S-1 in stage IIIA, B, C. (A) Stage IIIA, (B) Stage IIIB, (C) Stage IIIC.

**Additional file 2: Figure S2.** DFS and OS of XELOX and S-1 in N1, 2, 3. (A) N1 (B) N2 (C) N3.

**Additional file 3: Figure S3.** DFS and OS of XELOX and S-1 in LNR1, 2, 3. (A) LNR1 (B) LNR2 (C) LNR3.

## Data Availability

The data that support the findings of this study are available from the corresponding author but restrictions apply to the availability of these data, which were used under license for the current study, and so are not publicly available. Data are however available from the corresponding author upon reasonable request and with permission of Institutional Review Board of the Seoul St. Mary’s Hospital.

## Abbreviations

AJCC: American Joint Committee on Cancer
CA 19–9: Carbohydrate antigen 19–9
CEA: Carcinoembryonic antigen
CI: Confidence interval
CT: Computed tomography
DFS: Disease-free survival
ECOG: **Eastern Cooperative Oncology Group**
EGJ: Esophagogastric junction
HR: Hazard ratio
IQR: Interquartile range
LN: Lymph node
LNR: Lymph node ratio
MRI: Magnetic resonance imaging
OS: Overall survival
PSM: Propensity score matching
XELOX: Capecitabine and oxaliplatin
