# Supplementary figures and images for: Efficacy of capecitabine and oxaliplatin versus S-1 as adjuvant chemotherapy in gastric cancer after D2 lymph node dissection according to lymph node ratio and N stage
Source: BMC Cancer. 2019 Dec 18;19:1232. doi: 10.1186/s12885-019-6433-3 (PMC6921502; doi:10.1186/s12885-019-6433-3)

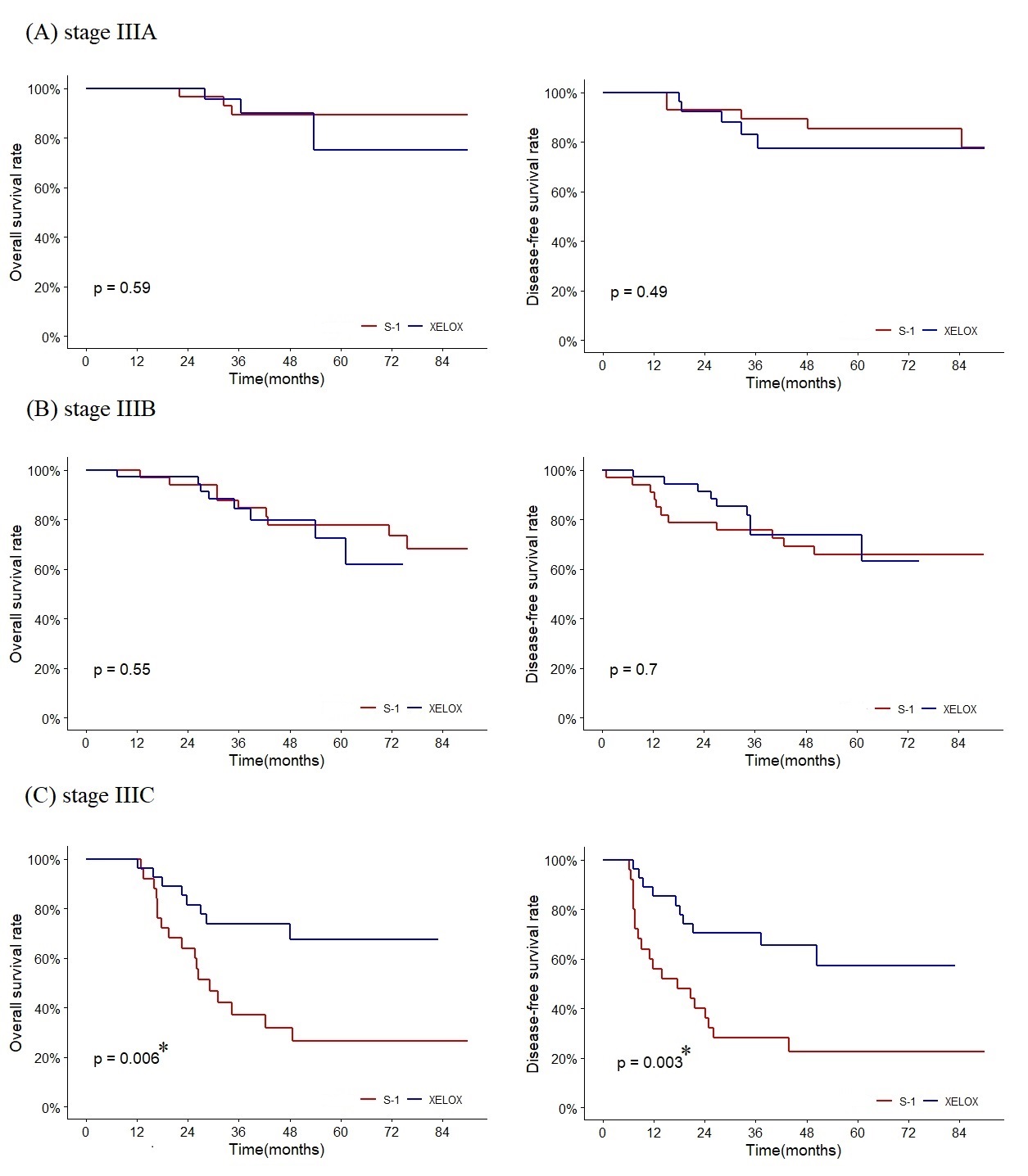

Supplement: Supplementary file 1 — Additional file 1: Figure S1. DFS and OS of XELOX and S-1 in stage IIIA, B, C. (A) Stage IIIA, (B) Stage IIIB, (C) Stage IIIC. [file 12885_2019_6433_MOESM1_ESM.jpg]

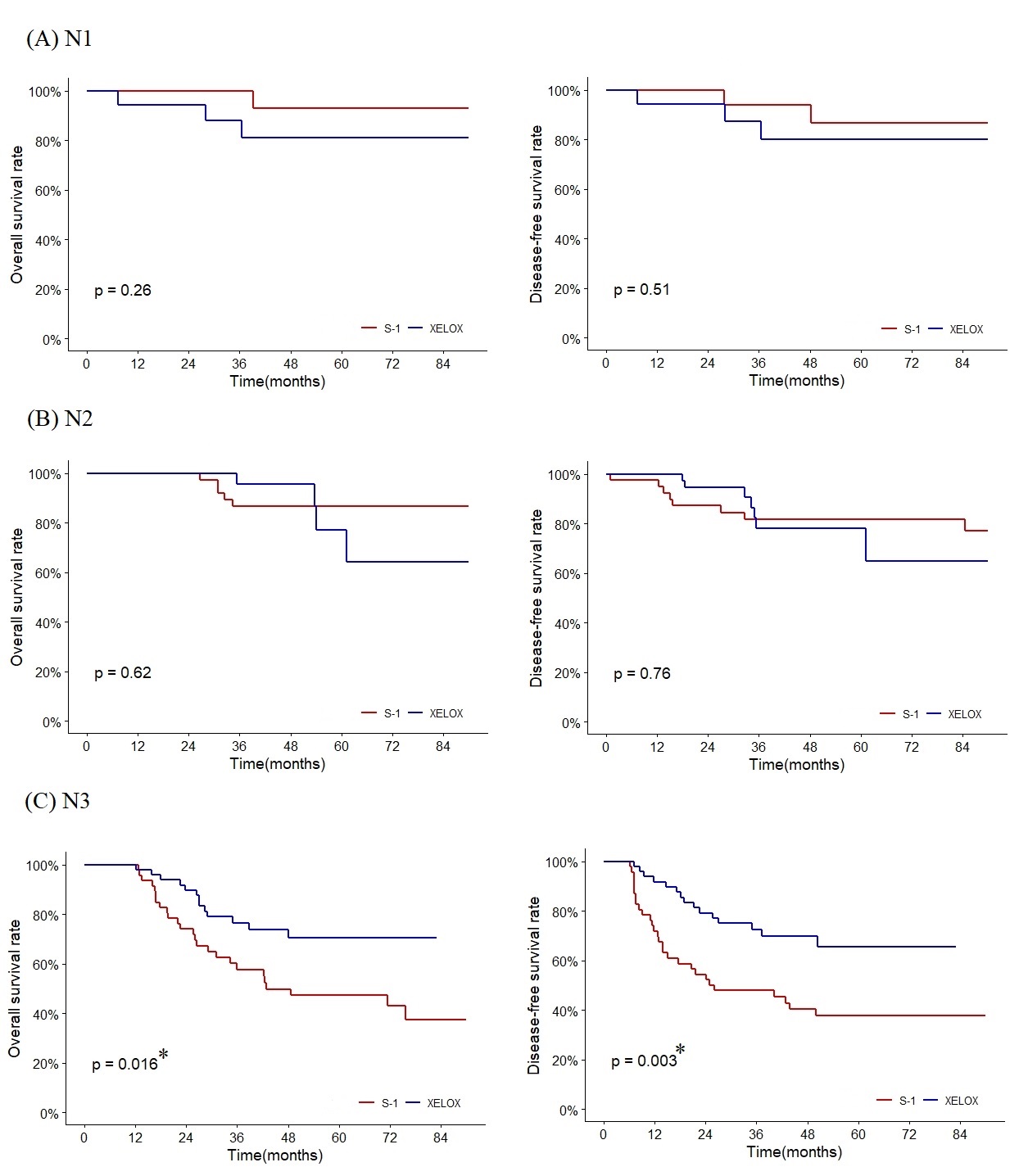

Supplement: Supplementary file 2 — Additional file 2: Figure S2. DFS and OS of XELOX and S-1 in N1, 2, 3. (A) N1 (B) N2 (C) N3. [file 12885_2019_6433_MOESM2_ESM.jpg]

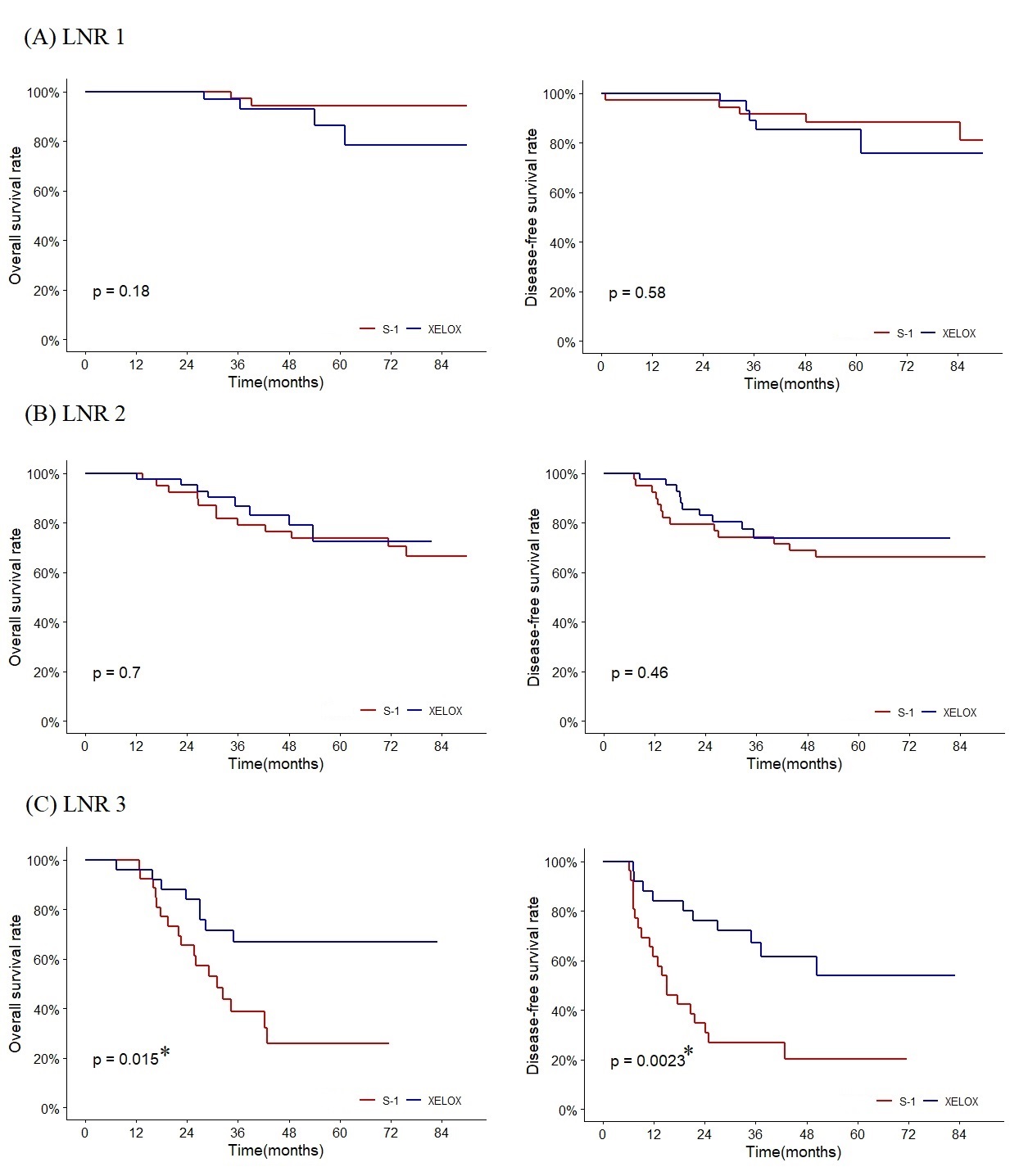

Supplement: Supplementary file 3 — Additional file 3: Figure S3. DFS and OS of XELOX and S-1 in LNR1, 2, 3. (A) LNR1 (B) LNR2 (C) LNR3. [file 12885_2019_6433_MOESM3_ESM.jpg]
